# Percutaneous hepatic perfusion with melphalan in uveal melanoma: A safe and effective treatment modality in an orphan disease

**DOI:** 10.1002/jso.24956

**Published:** 2017-12-28

**Authors:** Ioannis Karydis, Alexandra Gangi, Matthew J. Wheater, Junsung Choi, Iain Wilson, Kerry Thomas, Neil Pearce, Arjun Takhar, Sanjay Gupta, Danielle Hardman, Sean Sileno, Brian Stedman, Jonathan S. Zager, Christian Ottensmeier

**Affiliations:** ^1^ Cancer Sciences Academic Unit University of Southampton Southampton United Kingdom; ^2^ University Hospital Southampton Southampton United Kingdom; ^3^ Department of Cutaneous Oncology Moffitt Cancer Center Tampa Florida; ^4^ Department of Radiology Moffitt Cancer Center Tampa Florida; ^5^ Morsani School of Medicine University of South Florida Tampa Florida

**Keywords:** chemosaturation, intrahepatic percutaneous haemoperfusion, liver metastasis, melphalan, unresectable liver tumor, uveal melanoma

## Abstract

**Background:**

Metastatic uveal melanoma (UM) carries a poor prognosis; liver is the most frequent and often solitary site of recurrence. Available systemic treatments have not improved outcomes. Melphalan percutaneous hepatic perfusion (M‐PHP) allows selective intrahepatic delivery of high dose cytotoxic chemotherapy.

**Methods:**

Retrospective analysis of outcomes data of UM patients receiving M‐PHP at two institutions was performed. Tumor response and toxicity were evaluated using RECIST 1.1 and Common Terminology Criteria for Adverse Events (CTCAE) v4.03, respectively.

**Results:**

A total of 51 patients received 134 M‐PHP procedures (median of 2 M‐PHPs). 25 (49%) achieved a partial (*N* = 22, 43.1%) or complete hepatic response (*N* = 3, 5.9%). In 17 (33.3%) additional patients, the disease stabilized for at least 3 months, for a hepatic disease control rate of 82.4%. After median follow‐up of 367 days, median overall progression free (PFS) and hepatic progression free survival (hPFS) was 8.1 and 9.1 months, respectively and median overall survival was 15.3 months. There were no treatment related fatalities. Non‐hematologic grade 3‐4 events were seen in 19 (37.5%) patients and were mainly coagulopathic (*N* = 8) and cardiovascular (*N* = 9).

**Conclusions:**

M‐PHP results in durable intrahepatic disease control and can form the basis for an integrated multimodality treatment approach in appropriately selected UM patients.

## INTRODUCTION

Metastatic uveal melanoma (UM) carries a dismal prognosis with 1 year survival rates reported at 10‐25%.^1^, ^2^, ^3^ Unlike cutaneous melanoma (CM) where recent developments in the field of immunotherapy^4^ and targeted therapy^5^ have transformed the outlook in the metastatic setting, there are no established effective systemic treatments for metastatic UM. Activating BRAF mutations are rare^6^ and so far no alternative molecular targeted agents have demonstrated significant activity.^7^ Immunotherapy of UM to date has been extremely disappointing with response rates of <10%, much lower than those seen in CM.^8^, ^9^, ^10^ This is especially true in the context of progressive liver disease,^11^ which is common in metastatic UM as the liver is involved in >85% of cases of metastatic spread.^3^

The liver microenvironment is known to facilitate immune escape^12^ and the specific mechanisms involved may account both for the predilection of UM for liver metastases and the reduced efficacy of immunotherapeutic agents in patients with progressive liver disease. As the liver is the sole site of metastatic involvement in around 50% of UM cases,^3^ adopting a liver‐directed treatment approach can result in clinically meaningful periods of disease control while minimizing systemic toxicity. Resection or ablation of metastatic deposits is associated with prolonged survival in preselected patient groups.^13^, ^14^

For unresectable or multifocal small volume disease, arterially delivered methodsm—such as chemo‐, radio‐, and immunoembolization and isolated hepatic perfusion (IHP) have been devised.^15^ The concept is to exploit the differential blood supply to the metastatic deposits, derived almost exclusively from the hepatic artery,^16^ as opposed to the supply of the healthy parenchyma primarily from the portal vein.^17^ IHP was established as a surgical procedure involving temporary surgical isolation of the hepatic circulation and delivery of high dose of cytotoxic chemotherapy through the hepatic artery.^18^ While IHP was shown to have response rates of approximately 40‐50% in UM,^19^ the associated complexity, mortality, morbidity, and inability to retreat patients were significant drawbacks in early reports.

By utilizing advanced endovascular techniques, Percutaneous Hepatic Perfusion (PHP) significantly improves on this concept. A Phase I trial^20^ demonstrated the feasibility of this approach and a Phase III trial^21^ revealed a significantly improved primary end point of hepatic progression free survival (hPFS) as well as a significantly improved overall progression free survival (PFS) against best alternative care. Median overall survival (OS) was not significantly different between the groups but a high crossover rate (58%) made the effect on survival difficult to interpret.

In 2010, PHP became available for use at our institutions while a second phase III trial was planned. We present our multicentre experience with M‐PHP and compare our results with the original outcomes for UM patients in the Phase III study to investigate the safety and feasibility of delivering this technique outside a clinical trial setting.

## METHODS

### Patient eligibility

All patients with histologically confirmed UM who underwent M‐PHP in our institutions between December 2008 and October 2016 were included in this retrospective study. Approval for retrospective analysis of treatment outcomes was obtained from the institutional review boards of participating centres. Previous systemic or liver‐directed treatments other than M‐PHP were allowed provided any related adverse events (AEs) had either resolved or were not expected to impact the safety or efficacy of the procedure. Patients with known or suspected extrahepatic disease were included if disease was non‐progressive following previous treatments or amenable to ablative treatment modalities.

Generally, several weeks prior to M‐PHP, angiography is performed to delineate the arterial supply to the liver and a strategy for chemotherapy infusion is formulated. Occasionally coil embolization of vascular variants, such as the gastruoduodenal or right gastric arteries, that may predispose the patient to inadvertent flow of chemotherapeutic drugs into branches supplying the gastrointestinal tract may be required.

### Procedure

M‐PHP procedures were performed under general anaesthesia and with systemic anticoagulation in the interventional radiology suite. The patient has an arterial line, triple lumen catheter, and foley catheter placed for monitoring of arterial pressure, central venous pressure, and fluid management. The contralateral internal jugular vein (IJV) is accessed with a 10‐F vascular sheath, the common femoral (CFV) vein with a 18F sheath and the common femoral artery (CFA) with a 5 F sheath. After all lines are placed, the patient is anticoagulated with an initial dose of 300 U/kg of heparin and an activated clotting time (ACT) of ≥400 s is maintained throughout the procedure. Hepatic angiograms are obtained and the tip of a microcatheter is placed into the hepatic artery at the intended location of infusion. After placement of the infusion catheter in the hepatic artery, a 16‐F double‐balloon catheter (Delcath Systems Inc, New York, NY) is inserted via the CFV and positioned with its tip in the right atrium.

**Figure 1:**
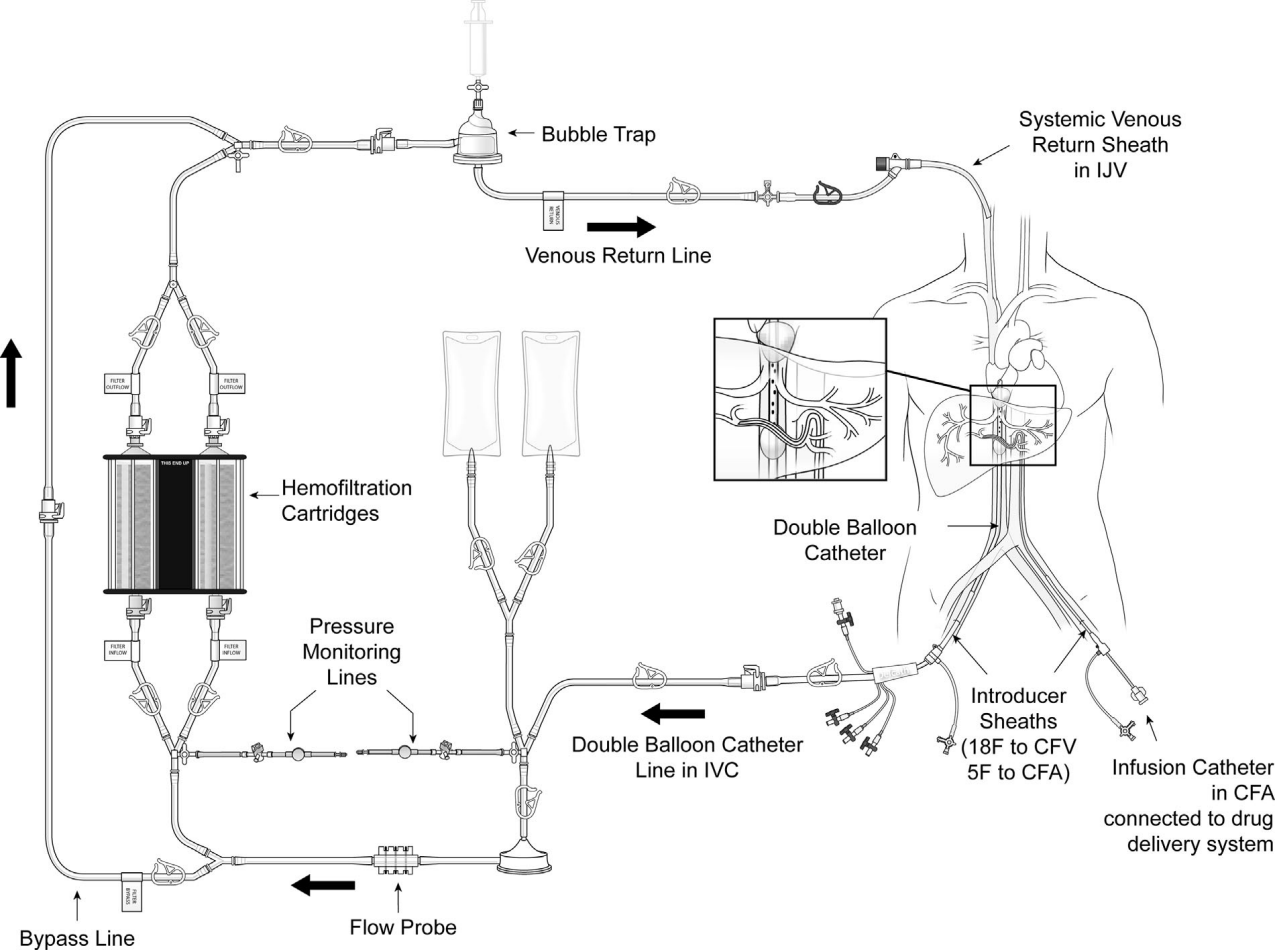
M‐PHP circuit

The catheter is then connected to an extracorporeal circulation system consisting of a centrifugal pump and two drug filtration activated carbon filters (Figure 1). Blood is aspirated through catheter fenestrations in a segment between the two balloons, actively pumped through the filtration system and returned through the sheath in the IJV. The cranial balloon of the catheter is inflated in the right atrium and retracted into the inferior vena cava (IVC). The caudal balloon is inflated in the IVC below the level of the hepatic veins and above the level of the renal veins. With both balloons inflated, a venogram is obtained to assess catheter position. With adequate positioning of the double‐balloon catheter, flow of the effluent hepatovenous blood to the systemic circulation is prevented by the cranial balloon at the atriocaval junction and by the caudal balloon at the level of the retrohepatic IVC.

Once correct positioning of the two balloons is confirmed, the filtration of blood by the two cartridges is started in a stepwise fashion. A centrifugal pump is used to achieve appropriate flow rates. The hemofiltration filters are brought online and once the cartridges are completely filled with blood, the bypass line is closed. When the hemofiltration circuit is running adequately and the patient is hemodynamically stable intra‐arterial infusion of melphalan is started. The dose of melphalan was calculated at 3 mg/kg, corrected for the patient's ideal body weight (maximum dose: 220 mg). After the infusion, extracorporeal filtration is continued for a 30 min (“washout period”) to allow clearance of melphalan from the liver.^20^ Post M‐PHP procedure, protamine sulphate is infused to reverse heparinization, and blood products are transfused to replace clotting factors as needed. The vascular sheaths are left in place until coagulation is sufficiently corrected. Once the patient coagulation profile normalizes, the vascular sheaths are removed and pressure is held on the sites for 45 min. Once stable, patients were transferred to the intensive care unit for monitoring and most received G‐CSF within 72 h of melphalan administration.

### Follow‐up

After hospital discharge patients had blood tests 1‐2×/week for up to 4 weeks to monitor hepatic function and full blood count; blood and platelet transfusions were arranged if necessary. Repeat imaging was arranged at 6‐12 week intervals and further PHP sessions were scheduled if there was no radiological evidence of intra‐ or extrahepatic disease progression, treatment was well tolerated, and treatment‐related toxicities resolved.

Repeat M‐PHP procedures were planned at approximately 8‐week intervals. The exact number of treatments is dependent on individual patients’ circumstances and local resource availability. In this patient cohort, patients treated in Southampton received up to four treatments while those treated at the Moffitt Cancer Center received up to six treatments. Also, if disease progression was felt to be attributable to differential perfusion of liver parenchyma due to anatomic constraints, subsequent attempts with M‐PHP would be made to preferentially target these areas.

### Response assessment

Either a dedicated liver MRI or a triple phase liver CT was performed to assess tumor response following the guidelines set forth in RECIST 1.1.^22^

### Data capture and analysis

Data was collected retrospectively from the electronic medical record. GraphPad Prism Version 6.01 was used for survival curve graphing and analysis using the Kaplan‐Meier method; log‐rank test was used to compare curves and determine the P value. SPSS version 23.0.0 was used for Cox regression analysis.

## RESULTS

### Patient characteristics

Fifty‐one patients with metastatic UM commenced M‐PHP between December of 2008 and October of 2016 at our two centres. All patients had pathologically confirmed metastatic UM to the liver and radiologically confirmed hepatic progression; 8/51 patients (15.7%) also had limited extrahepatic disease. Baseline patient characteristics are presented in Table 1.

**Table 1:** Baseline patient characteristics

A. Demographics		
Median age at 1st treatment (range)	57.9 years	(27.9‐77.1)
Median time to treatment from diagnosis of stage IV disease (range)	139 days	(30‐800)
Gender		
Female	28	(54.9%)
Male	23	(45.1%)

B. Disease extent at treatment onset		
Intrahepatic only	43	(84.3%)
Oligometastatic liver disease (< = 3 deposits)	12	(23.5%)
Heavy intrahepatic disease burden (>10 lesions/>50% volume replacement)	16	(31.4%)

C. Potential adverse outcome indicators		
Transaminitis at treatment onset	17	(33.3%)
LDH outside normal limits	19	(50.0%)^a^
PS > 0	6	(11.8%)

D. Previous treatment modalities		
Any previous liver directed treatment modalities	14	(27.5%)
Resections /ablations	9	(17.6%)
TACE/SIRT	9	(17.6%)
Previous systemic treatments	15	(29.4%)
Immunotherapy^b^	15	(29.4%)
Chemotherapy (dacarbazine)	2	(3.9%)
Clinical trial	1	(2.0%)

LDH, Lactate dehydrogenase; PS, Performance Status; TACE, transarterial chemoembolization; SIRT, Selective internal radiation therapy.

^a^ Denominator used is number of patients with available data for LDH at baseline (*N* = 38).

^b^ Ten ipilimumab; three pembrolizumab; two ipilimumab/nivolumab combination.

All patients underwent at least 1 M‐PHP. At data collection cut‐off time, two patients were lost to follow‐up and 17 patients were still alive; a median of two cycles of M‐PHP had been administered per patient, 134 M‐PHPs in total. Of these, seven patients were continuing on treatment, and 15 patients had completed the planned full course. Twenty‐nine patients discontinued early; nine due to treatment related toxicity, 17 due to disease progression, and three due to patient preference.

### Response analysis

**Figure 2:**
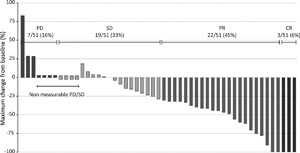
Waterfall plot of the best objective hepatic response to M‐PHP, measured as the maximum change from baseline in the sum of the longest diameter of each liver target lesion

Radiological assessments took place as clinically indicated, typically 6‐8 weeks after each treatment. Table 2A summarizes response outcomes: radiographic hepatic complete response (CR) was seen in 3/51 (5.9%) patients and radiographic partial hepatic response (PR) in 22/51 (43.1%) for an overall hepatic response rate (hORR) of 49%. Overall response rate (ORR) was 24/51 (47.0%) as one patient exhibited a hepatic response but progressed systemically at the time of the first assessment. Figure 2 illustrates by way of a waterfall plot the magnitude of observed responses.

**Table 2:** response rates by RECIST 1.1 criteria

a. Best overall and hepatic response in entire patient population				
	*N *= Percentage		*N *= Percentage	
	Overall		Hepatic	
CR	2	3.9%	3	5.9%
PR	22	43.1%	22	43.1%
SD	19	37.2%	19	37.2%
>3 months	16	31.3%	17	33.3%
>6 months	10	19.6%	11	21.6%
PD	8	15.6%	7	13.7%
Total assessable patients	51		51	

b. Best Hepatic Response by disease burden^a^				
	Low disease burden		High disease burden	
CR/PR	18	51.4%	7	43.7%
SD	11	31.4%	8	50.0%
PD	6	17.1%	1	6.3%
Total assessable patients	35		16	

CR, complete Response; PR, partial response; SD, stable disease; PD, progressive disease.

^a^ “high“ disease burden implies more than 10 lesions or more than 50% parenchymal involvement.

In 17/51 (33.3%) patients, the best hepatic response was stable disease (SD) for a minimum of three months; in 11 this was maintained for more than six months. Six month overall and hepatic disease control rates were 64.7% and 70.6%, respectively as there were three patients who progressed systemically despite ongoing disease response in the liver. There was no significant difference in response rates according to extent of intrahepatic disease (Table 2B, *P* = 0.35).

First site of disease progression on/after M‐PHP was known in 41 out of 43 patients who had progressed at the time of data cut off; in 18/41(43.9%) only the liver was involved while in 13/41 (31%) progression was exclusively in extrahepatic sites. There was extrahepatic involvement in 18/35 (51.4%) of patients with liver only disease at baseline as opposed to 5/6 (83%) of those with evidence of extrahepatic disease on treatment onset.

### Survival analysis

**Figure 3:**
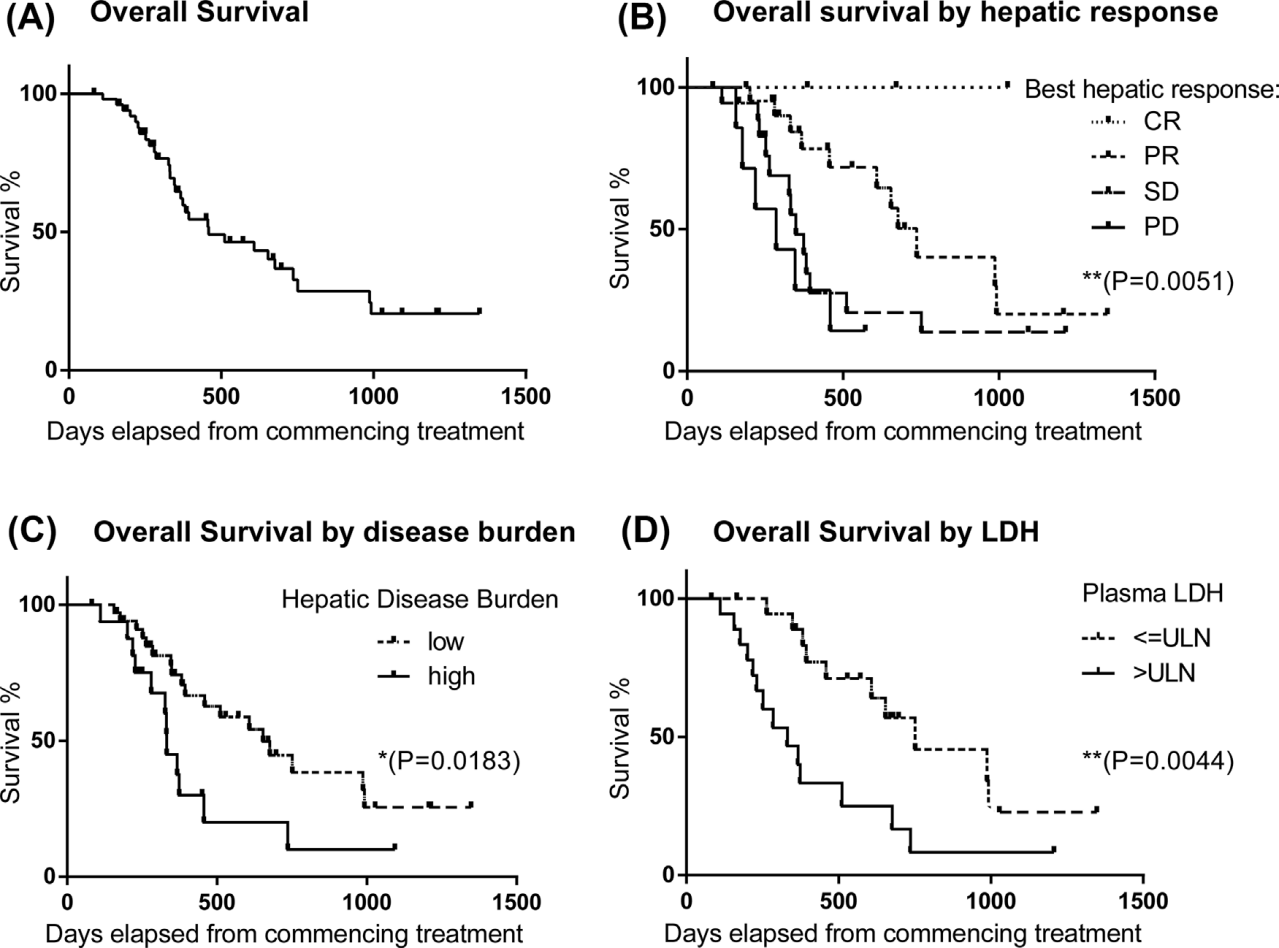
Kaplan‐Meier plots of overall survival of UM patients treated with M‐PHP. (A) Curve for entire group. (B‐D) Curves stratified by best response to M‐PHP (B), disease burden at baseline (C) and serum LDH (D)

After a median follow‐up of 12.2 months, at the time of data cut off, median OS was 15.3 months. Seventeen patients were still alive, five on‐treatment, 32 had passed away and two were lost to follow‐up. One year OS rate is 64.6% (Figure 3A). Patients who responded had significantly improved survival as opposed to non‐responders (Figure 3B, *P* < 0.01). Two‐year OS for the responders was 50.2% versus 18.8% in non‐responders.

On univariate analysis only high baseline LDH, high disease burden (50% liver parenchymal replacement and/or >10 deposits) and presence of extrahepatic disease at treatment onset predicted for worse OS (Figure 3C–E) while age, gender, previous liver directed, or systemic treatment, prolonged lead time from diagnosis of stage IV disease, ECOG Performance status, and deranged baseline liver function, did not.

**Figure 4:**
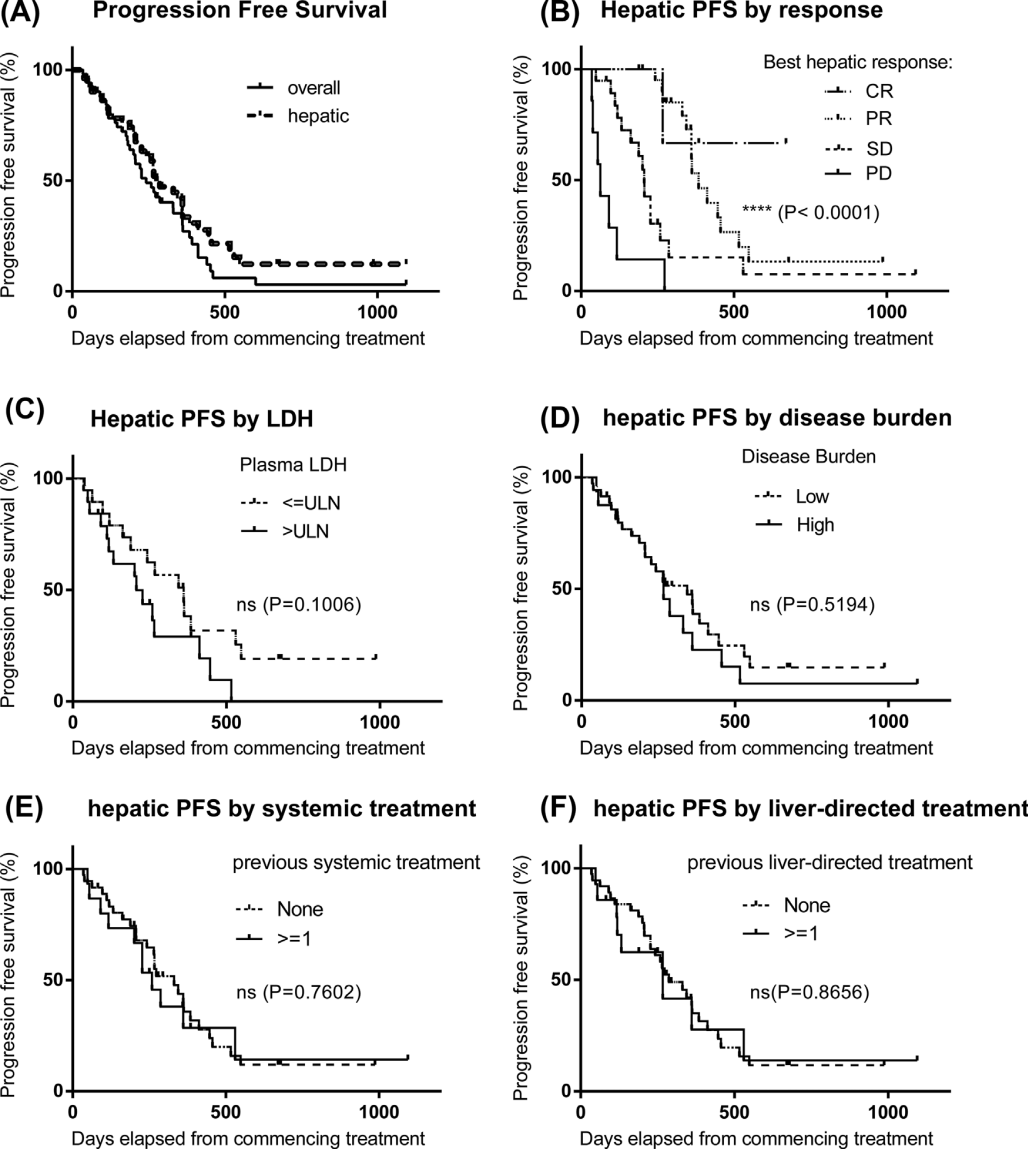
Kaplan–Meier plots of overall and hepatic progression free survival of UM patients treated with M‐PHP. (A) Curves for entire group, median PFS and hPFS not reached. (B‐F) Curves stratified by best response to M‐PHP (B), serum LDH (B), disease burden at baseline (D) and previous systemic (E) or liver‐directed (F) treatment

Overall PFS and hPFS were 8.1 and 9.1 months, respectively (Figure 4A). One year hPFS rates were 58.5% for responders and 15.1% for patients with stable disease (Figure 4B). Disease burden, serum LDH, previous liver directed, or systemic treatment did not influence hPFS on univariate analysis (Figure 4C–F); presence of extrahepatic disease at baseline was of borderline significance for both overall PFS and hPFS (*P *< 0.05 by Gehan‐Breslow‐Wilcoxon but not by log Rank test).

### Safety analysis

M‐PHP was well tolerated in this study population with frequency and adverse events types commensurate with those reported in the original Phase III study^21^ (Table 3). There were no treatment related fatalities. Nineteen patients (37.5%) experienced grade 3‐4 non‐hematologic treatment related toxicity. Cardiovascular toxicity was primarily observed peri‐procedurally—three cases of ventricular tachycardia and one case of supraventricular tachycardia were seen. There were five cases of post‐operative troponin elevation, one with associated ECG changes suggestive of non‐ST elevation myocardial infarction and one associated with pulmonary oedema. In addition there were two episodes of pulmonary oedema without documented associated myocardial ischaemia.

**Table 3:** Treatment related adverse events seen in patients receiving at least one M‐PHP procedure

Adverse event	Any grade *N* = %		Grade 3‐4 *N* = %	
Hematological toxicity				
Anemia	51	100.0%	15	29.4%
Neutropenia	22	43.1%	16	31.3%
Thrombocytopenia	50	98.0%	16	31.3%
Coagulopathic toxicity				
Hemorrhagic Event	10	19.6%	2	3.9%
Thromboembolic Event	7	13.7%	6	11.8%
Cardiovascular toxicity				
Any	11	21.6%	9	17.6%
Arrhythmias	5	9.8%	4	7.8%
Pulmonary Edema	3	5.9%	3	5.9%
Cardiac Ischemia	5	9.8%	5	9.8%
Cerebrovascular event	2	3.9%	0	0.0%
Late toxicity				
Fatigue	17	33.3%	1	2.0%
Mucositis	1	2.0%	0	0.0%
Nausea	12	23.5%	0	0.0%
Vomiting	8	15.6%	0	0.0%
Epigastric pain	6	11.8%	0	0.0%
Transaminitis	15	29.4%	3	5.9%
Rash	1	2.0%	0	0.0%
Constipation	2	3.9%	0	0.0%

Bleeding events were common peri‐operatively and seen in 19.6% of patients, but most were minor. There was 1 case each of DIC requiring prolonged clotting factor support, intra‐abdominal bleeding, and intracerebral haemorrhage—not tumor related—all resolved with no long term sequelae.

Thromboembolic events were a notable intermediate/late complication in 13.7% of patients—two cases of pulmonary embolism, one each of inferior vena cava, left internal jugular, and vascular access site‐related thrombus, and two lower limb DVTs were reported within 2 months of a PHP procedure.

Immediate post‐procedure (within 24 h of M‐PHP) haematological toxicities were common with grade 3‐4 thrombocytopenia and anaemia seen in 27.4% and 31.4% of patients, respectively. Twenty‐four patients (47.1%) received a RBC transfusion and 40 (78.4%) received a platelet transfusion. Late neutropenia was seen in 43.1% despite routine G‐CSF support—16 patients (31.4%) experienced at least one episode of grade three neutropenia, but there were only four documented episodes of neutropenic sepsis.

Transaminitis was seen in 29.4% of patients but was typically mild and resolved rapidly (within 1‐2 weeks) after the procedure in almost all the cases. Only 5.9% of patients experienced grade 3‐4 events. Other AEs likely relating to systemic escape of melphalan were mild and self‐limiting and are summarized in Table 3.

## DISCUSSION

This retrospective analysis of UM patients treated with M‐PHP at two institutions demonstrates that M‐PHP can be administered safely and effectively in high volume treatment centres in appropriately selected patients. Additionally, toxicity rates are comparable to those reported in the phase III trial^21^: cardiovascular toxicity was seen in nine patients (17.6%) versus 12 (17%) in the phase III trial; severe neutropenia was seen in 16 (31.4%) patients as opposed to 60 (85.7%), and there were only four cases (7.8%) of febrile neutropenia as opposed to 12 (17.1%). Importantly, while the trial PHP related mortality was 6% (4/70 patients), in our series there were no treatment related deaths.

The explanation for improved safety outcomes is likely multifactorial. Our patients were treated in high volume centres—carrying out more than six procedures per year—by experienced teams. Patient selection criteria were strict: patients with known extrahepatic disease were only offered treatment if it was amenable to resection or ablation. Physiological fitness was formally assessed by experienced intensivists, only one patient treated had a history of cardio‐ or cerebrovascular disease—a transient ischaemic attack 5 years prior to the first treatment—and none had a known history of bleeding or pro‐thrombotic tendencies All patients had an ECOG performance status of 1 or better. Patients with heavy disease burden had to have preserved hepatic function.

A second generation filter was used that might have contributed to increased melphalan extraction, reducing late bone marrow suppression. Finally, the median number of cycles of M‐PHP received was lower than in the trial, largely due to logistical issues at one of the treatment centres—a median of two cycles at UHS versus 3 at Moffitt Cancer Center and the phase III trial.

Despite the lower number of administered procedures per patient, hepatic responses were seen in a similar proportion of patients: 49% in the current study versus 36% in the previous phase III trial. Additionally, the current study demonstrated high hepatic disease control rates; median hPFS was at 9.1 months in the current report versus 8.2 months in the trial. Most importantly, OS rates were encouraging when compared with historical data—64.6% at 1 year in the current report compared to 38% in the trial. Two year overall survival rates in our series was 36.8%, and could further be broken down to 56.0% in responders and 20.6% in those with stable disease.

This apparent improvement may be partly attributable to differences in patient selection. Our series only includes UM patients of whom only 9 (17.6%) had extrahepatic disease at the initiation of treatment and in almost all cases this was either quiescent, treatable with ablative modalities or resected between M‐PHPs. In 42.9% (3/7) of these patients who progressed this was with a new site of extrahepatic disease only as opposed to 26.4% (9/34) of patients with intrahepatic only disease and extrahepatic disease was associated with worse outcomes (Figure 2E). The proportion of patients with extrahepatic disease in the phase III trial was close to 40% and 10% of patients had a diagnosis of metastatic CM. Lastly, 30% of patients had an ECOG PS of 1 versus 12% in our series.

Another possibility relates to the recent advent of immunotherapy such as anti PD‐1 and anti‐CTLA‐4 agents that were not available at the time of the original trial. In CM, these have resulted in a dramatic improvement in patient outcomes,^4^ but their place in UM is much less certain as UM patients were excluded from the original phase III trials and in small case series results are disappointing.^10^, ^11^ In our series 31 (60.7%) of the patients went on to receive immunotherapy after completing a course of PHP; 20 received ipilimumab, 21 pembrolizumab, and three other experimental immunotherapeutic approaches.

In a recently published case series of UM patients treated with second line pembrolizumab, outcomes were much better for patients without progressive liver‐only disease.^11^ It is possible that in our group of patients, the degree of intrahepatic disease control provided by M‐PHP was sufficient to augment the systemic effect of immunotherapy, and at least partially overcome UM's innate resistance to this treatment modality. It is conceivable that controlling rapidly progressive intrahepatic disease simply provides the immune system with more time to mount a response when augmented by immune‐checkpoint inhibitors. Release of tumor antigens and modification of an immunosuppressive liver microenvironment are additional ways through which PHP might augment an anti‐tumor immune response. The debulking effect may also further delay systemic disease spread by reducing the source of viable circulating tumor cells.

Finally, we need to consider that improved outcomes may simply be a “stage migration”‐like effect due to selection of patients with earlier disease. Historically presentation was late and driven by chance findings of deranged liver function tests or symptoms relating to liver capsule pain or biliary tract obstruction. As more treatment modalities are becoming available, there has been increased recognition of the importance of early diagnosis and routine biannual liver imaging in high‐risk patients is now considered standard practice.^23^

The debate regarding the merit of regional therapy in melanoma and in particular of liver directed therapy in UM is longstanding.^24^ It is unfortunate that the original phase III randomized control trial of M‐PHP versus best alternative care allowed for both large scale crossover and was not limited to UM thereby failing to demonstrate unequivocal OS benefit in this patient group.

Our results clearly demonstrate that M‐PHP appears to be an effective means of obtaining rapid intrahepatic disease control, is a sensible option in patients with liver predominant disease in the absence of established effective systemic treatments and support the role of M‐PHP as part of an integrated multi‐disciplinary approach to the management of UM. A phase III pivotal randomized study is underway to more robustly quantify the magnitude of benefit in specific subgroups and help establish how M‐PHP can be optimally placed in an integrated pathway of patients with advanced UM.

## CONCLUSION

Our results demonstrate that M‐PHP can be safely employed in appropriately selected UM patients with primarily liver based disease as part of an integrated multi‐disciplinary approach in institutions with appropriate expertise. Outcomes compare favorably to currently available treatment modalities, however further research is needed to determine optimal treatment strategies.

## CONFLICTS OF INTEREST

Dr Stedman has received honoraria for lecturing and has acted as a medical advisor to Delcath Systems Inc. Dr Karydis and Dr Ottensmeier have received a travel grant by Delcath Systems Inc. Dr Zager serves on the medical advisory board for Delcath Systems and has research funding from Delcath Systems. All remaining authors have declared no conflicts of interest.
